# Cost-Effectiveness of a National Initiative to Improve Hand Hygiene Compliance Using the Outcome of Healthcare Associated *Staphylococcus aureus* Bacteraemia

**DOI:** 10.1371/journal.pone.0148190

**Published:** 2016-02-09

**Authors:** Nicholas Graves, Katie Page, Elizabeth Martin, David Brain, Lisa Hall, Megan Campbell, Naomi Fulop, Nerina Jimmeison, Katherine White, David Paterson, Adrian G. Barnett

**Affiliations:** 1 Institute of Health & Biomedical Innovation, Queensland University of Technology, Brisbane, Queensland, Australia; 2 Department of Applied Health Research, University College London, London, United Kingdom; 3 School of Management, Queensland University of Technology, Brisbane, Australia; 4 Centre for Clinical Research, University of Queensland, Brisbane, Queensland, Australia; Cleveland Clinic, UNITED STATES

## Abstract

**Background:**

The objective is to estimate the incremental cost-effectiveness of the Australian National Hand Hygiene Inititiave implemented between 2009 and 2012 using healthcare associated *Staphylococcus aureus* bacteraemia as the outcome. Baseline comparators are the eight existing state and territory hand hygiene programmes. The setting is the Australian public healthcare system and 1,294,656 admissions from the 50 largest Australian hospitals are included.

**Methods:**

The design is a cost-effectiveness modelling study using a before and after quasi-experimental design. The primary outcome is cost per life year saved from reduced cases of healthcare associated *Staphylococcus aureus* bacteraemia, with cost estimated by the annual on-going maintenance costs less the costs saved from fewer infections. Data were harvested from existing sources or were collected prospectively and the time horizon for the model was 12 months, 2011–2012.

**Findings:**

No useable pre-implementation *Staphylococcus aureus* bacteraemia data were made available from the 11 study hospitals in Victoria or the single hospital in Northern Territory leaving 38 hospitals among six states and territories available for cost-effectiveness analyses. Total annual costs increased by $2,851,475 for a return of 96 years of life giving an incremental cost-effectiveness ratio (ICER) of $29,700 per life year gained. Probabilistic sensitivity analysis revealed a 100% chance the initiative was cost effective in the Australian Capital Territory and Queensland, with ICERs of $1,030 and $8,988 respectively. There was an 81% chance it was cost effective in New South Wales with an ICER of $33,353, a 26% chance for South Australia with an ICER of $64,729 and a 1% chance for Tasmania and Western Australia. The 12 hospitals in Victoria and the Northern Territory incur annual on-going maintenance costs of $1.51M; no information was available to describe cost savings or health benefits.

**Conclusions:**

The Australian National Hand Hygiene Initiative was cost-effective against an Australian threshold of $42,000 per life year gained. The return on investment varied among the states and territories of Australia.

## Introduction

Improved compliance to hand hygiene among healthcare workers will likely contribute to lower rates of healthcare associated infection [1] and improve patient outcomes. Interest in hand hygiene increased with the patient safety movement that emerged in the 1990s [2] and hospitals today are on average safer places than before. The World Health Organisation is leading a global effort to improve hand hygiene compliance with 170 nations signed up to the ‘Clean Care is Safer Care’ campaign. Allegranzi *et al*. [3] have shown compliance in Costa Rica, Italy, Mali, Pakistan, and Saudi Arabia increased from 51.0% to 67.2%. A version of the WHO campaign was implemented in Germany where more than 700 healthcare institutions participated including 28 university hospitals. There was an absolute increase of 11% in hand hygiene compliance in 62 hospitals [4]. The experience in England and Wales for the period 2004 to 2008 among 187 trust hospitals [5] showed combined procurement of soap and alcohol hand rub tripled, rates fell for MRSA bacteraemia from 1.88 to 0.91 cases per 10,000 bed days, and for *C*. *difficile* infection from 16.75 to 9.49 cases per 10,000 bed days, but MSSA bacteraemia rates did not fall. Adjustments were not made for all national concurrent initiatives that would also likely affect MRSA and *C*. *difficile*.

Systematic reviews of the evidence for the effectiveness of hand hygiene programmes on hand hygiene compliance have been published [6–10] and two reported pooled results. Schweizer et al. [9] used evidence from three studies to describe a bundle of education, reminders, feedback, administrative support, and access to alcohol-based hand rub to show a pooled odds ratio of 1.82 (95% CI, 1.69–1.97). A further three studies were used to describe a bundle of education, reminders, and feedback that showed a pooled odds ratio of 1.47 (95% confidence interval 1.12–1.94). Luangasanatip et al. [10] did a meta-analysis of two randomised controlled trials and showed the addition of goal setting to the World Health Organization 2005 campaign (WHO-5) was associated with improved compliance (pooled odds ratio 1.35 (95% confidence interval 1.04 to 1.76).

The evidence base for the effectiveness of interventions to improve hand hygiene is growing and programmes are being rolled out globally. Our knowledge of whether these interventions are good value for money is limited. Infection prevention is traditionally underfunded with infection prevention departments seen as cost centres rather than flagship clinical services [11]. Obtaining the largest health returns from scarce infection prevention budgets should be a priority. How improving hand hygiene compliance fits with other infection prevention activities is not well understood [12]. Investing to further improve hand hygiene compliance might displace other infection prevention efforts such as prospective surveillance, screening and isolating patients, ensuring prophylaxis for surgery is done appropriately, environmental cleaning and antimicrobial stewardship programmes. A balanced portfolio of infection prevention activities that generates the largest health return per dollar invested should be a policy goal [13, 14].

A cost-effectiveness analysis will reveal how improving hygiene compliance impacts on costs and health benefits [13]. These studies report a cost per life year gained and so summarise the value for money of a programme and allow comparison with competing uses of scarce infection control resources [14]. If the health return is low per dollar invested then we might seek other ways to use the infection control budget. If the health return is large then investments in new hand hygiene compliance programmes are justified.

This research describes a national scale initiative funded by the Australian Commission on Safety and Quality in Health Care implemented between 2009 and 2012 in all states and territories of Australia. It was managed by Hand Hygiene Australia [15] and there were three stated goals. To achieve widespread adoption of the World Health Organisation’s ‘five moments’ programme [16]. To apply a single training programme that allows healthcare workers responsible for hand hygiene to teach and measure compliance in a similar way. To promote accurate measurement of new hospital-associated *Staphylococcus aureus* bacteraemia infections using a standard definition, suggested as a valid and sensitive outcome measure for hand hygiene [17, 18]. The national programme was designed to be simple to use and allow outcomes to be compared across Australia and internationally.

The national initiative augmented existing local efforts to improve hand hygiene compliance among the eight states and territories of Australia. In Queensland (QLD) there was an existing programme that promoted 11 tasks of hand hygiene [19], funded by the state health department and implemented in all QLD hospitals by the Centre for Healthcare Related Infection Surveillance and Prevention. Some data were reported that showed high compliance but there was little education for auditors and relatively few numbers of observations were recorded. In South Australia (SA) and Western Australia (WA) there were campaigns within single hospitals but no state wide implementation other than the health department providing support material such as posters. In Tasmania (TAS), Australian Capital Territory (ACT) and Northern Territory (NT) there were no hospital level or state level campaigns. In New South Wales (NSW) there was a state funded programme starting in 2006 [20]. It included education, measurement of alcohol based hand rub usage and auditing of hand hygiene behaviour with feedback to staff. In Victoria (VIC) the state health service funded a pilot programme at the Austin Hospital for six sites [21] that was subsequently expanded to a state wide programme [22]. Grayson et al [22] reported a statistically significant reduction in MRSA clinical isolates per 100 patient days of –0.018 per month (95% CI –0.024 to –0.011, p-value <0.001). This analysis incorrectly assumed rates were flat prior to implementation. A re-analysis of the same data that allowed an appropriate linear decrease in rates before the intervention showed effectiveness is reduced to –0.007 and is not statistically significant (95% CI –0.019 to 0.006, p-value = 0.31) [23]. Playford at el. [24] found counterintuitively that hand hygiene compliance and healthcare associated *Staphylococcus aureus* bacteraemia were positively correlated, and their data came from 21 large Australian metropolitan public hospitals. They caution against using healthcare associated *Staphylococcus aureus* bacteraemia as a sole indicator of the impact of HH compliance citing other important causal factors in the relationship.

The objective for this paper is to report the incremental change to costs and health outcomes relating to healthcare associated *Staphylococcus aureus* bacteraemia infections from a decision to adopt the National Hand Hygiene Initiative implemented between 2009 and 2012.

## Methods

The design was a cost-effectiveness modelling study to predict the change to the number of cases of healthcare associated *Staphylococcus aureus* bacteraemia, change to years of life lost among inpatient admissions, and change to total health services costs defined by the annual on-going maintenance costs less the cost savings from fewer cases of *Staphylococcus aureus* bacteraemia infection. Data have been collected for the National Hand Hygiene Initiative on compliance rates for each of the five hand hygiene moments since 2009. The number of moments recorded has increased from 127,125 in November 2009 to 461,082 in March 2013 and compliance rose from 61.8% to 76.9% during this time [25]. We chose not to use this measure an outcome for this study because: healthcare workers might only improve their hand hygiene while they are observed by auditors [26]; many of the data were missing; large discrepancies between the performance of nursing and medical staff will bias the reported averages [27]; and, most important is that reliable and complete outcomes were available for *Staphylococcus aureus* bacteraemia in the time periods before and after implementation of the National Hand Hygiene Initiative.

*Staphylococcus aureus* bacteraemia is the only infection outcome included for this study and the definition has been published [28], see Appendix A in S1 File. *Staphylococcus aureus* bacteraemia is the only healthcare associated infection with a nationally agreed definition among the eight jurisdictions of Australia [29]. Cases of *Staphylococcus aureus* bacteraemia have large costs arising from extra days stay in hospital required to treat symptoms [30] and so their reduction will show the largest possible cost savings. Acquisition also increases mortality risk substantially [30, 31] and quantifying reduction is an opportunity to show the health benefits of infection control in ‘years of life gained’, which is a suitable outcome measure for cost-effectiveness research [32]. The steering committee formed to oversee this cost-effectiveness evaluation made up of representatives of Hand Hygiene Australia, The Australian Commission on Safety and Quality in Health Care, and all the state and territory health departments strongly advised the research team not to include data on other infection outcomes, due to inconsistency in how they had been measured among the 8 state and territory health services. This is confirmed by recent evidence that shows great variability in surveillance practices among the eight states and territories [33].

**Fig 1:**
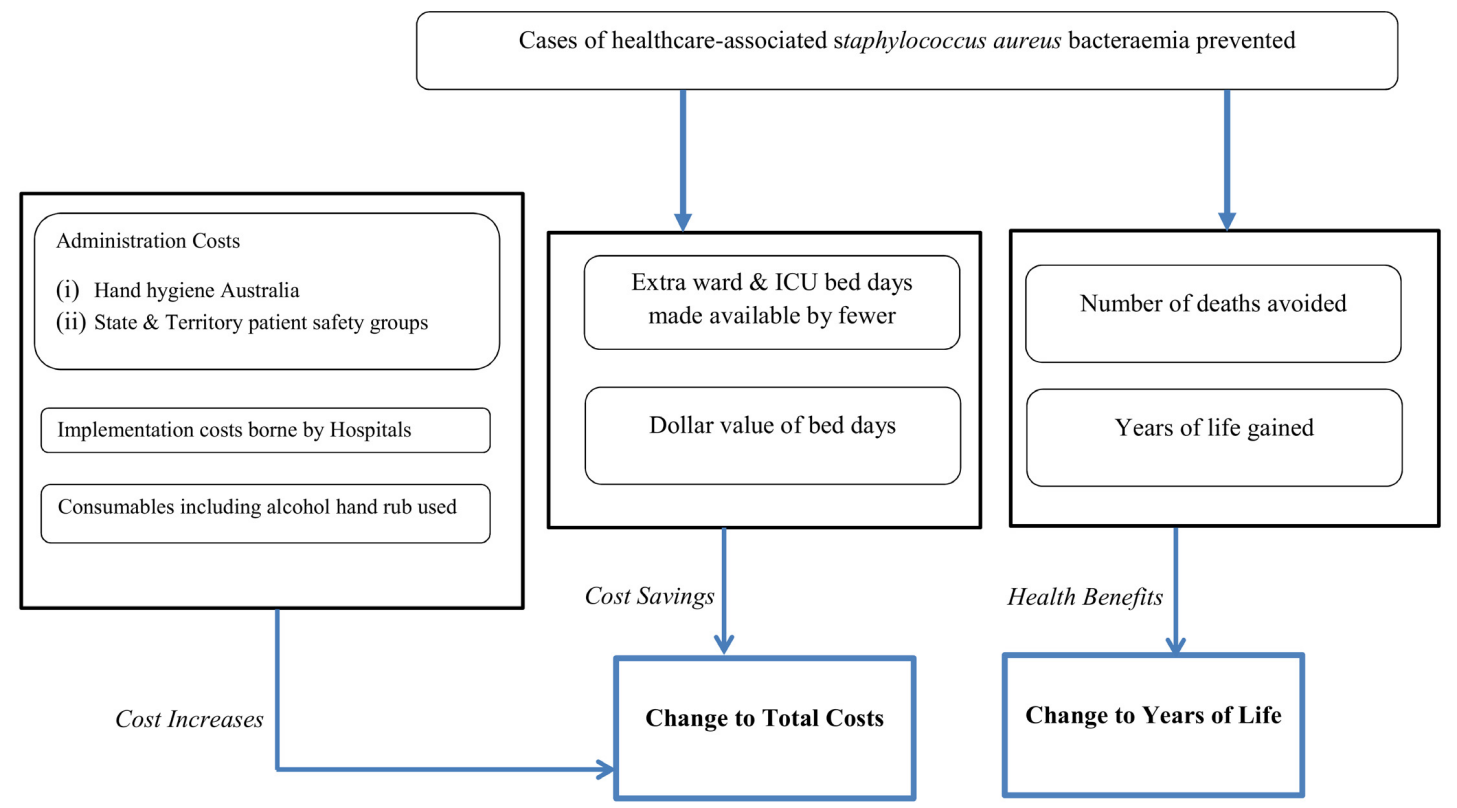
How information is structured to predict incremental cost-effectiveness outcomes.

Cost and effectiveness outcomes were estimated separately for each state and territory of Australia, using the comparator of the pre-existing and locally organised hand hygiene initiatives. The cost per life year gained was used to judge the cost-effectiveness of the national initiative and arose from dividing change to total cost by the change to life years gained. Total cost was the ongoing implementation costs less the cost savings from fewer cases of *Staphylococcus aureus* bacteraemia. The perspective adopted was acute services funded by the state and territory health department, because a decision to continue investing in hand hygiene programmes would be made at this level rather than nationally. Future costs and health benefits were discounted at 3% [34]. Model outcomes were evaluated for a 12-month period after the implementation of the National Hand Hygiene Initiative, 2011–2012. How information was structured to predict incremental cost-effectiveness is shown in Fig 1.

### Setting and Patients

Data were collected from the 50 largest acute public hospitals in Australia representative of the eight states and territories, Appendix B in S1 File. The sample was the five largest public hospitals by number of acute beds in NSW, VIC, QLD, WA and SA, the three largest public hospitals in TAS, and the single main public hospital in NT and the ACT; the next largest 20 public hospitals Australia-wide were then selected. These hospitals provide 42% of the public acute beds in Australia and suggest a sample that can be generalised from. There were 1,294,656 admissions to 24,482 beds included in the cost-effectiveness model for the 12-month period in 2011–2012, Table 1. No useable pre-intervention *Staphylococcus aureus* bacteraemia data were made available from 11 hospitals in VIC or the single hospital in NT. No private hospitals were included in the study.

### Evaluation of the impact of National Hand Hygiene Initiative on Staphylococcus aureus bacteraemia

Reductions in risk of *Staphylococcus aureus* bacteraemia from the introduction of the national initiative were estimated using 2,304 monthly infection rates across 38 hospitals in six states and territories. A brief summary is provided and a complete description of the statistical method and results are available [35]. There was variability in the start date among sites, Appendix C in S1 File. The average number of months for which data were available before the intervention was 29 per hospital, with an average post-intervention time of 32 months. A before and after quasi-experimental design [36] was used to compare rates of *Staphylococcus aureus* bacteraemia in pre and post-intervention time periods. Analyses were conducted separately for each state and territory because the intervention was implemented at slightly different times, there were important differences in average infection rates, pre-existing hand hygiene campaigns varied and there were different infection prevention policies. We controlled for seasonal patterns in rates using a categorical variable for month and we used a random intercept in each hospital to control for differences in the average infection rates between hospitals. We were not interested in differences between hospitals and estimated the within-hospital change due to the intervention, and the average within-hospital change per state.

**Table 1:** Information used to estimate the number of cases of Staphylococcus aureus *bacteraemia* (SAB) prevented by the National Hand Hygiene Initiative.

Patient cohort included in model				Baseline starting rates SAB *		Reduction in rates †		Relative risk of SAB †		Nature of the Decline
State/Territory	Hospitals	Beds	Admissions	Mean	St. Dev.	Mean	95% CI	Mean	St. Dev.	
QLD	9	5,366	246,699	1.48	1.08	17%	6 to 27%	0.83	0.10	Immediate reduction sustained over time
ACT	1	619	31,841	2.91	n/a	28%	6 to 45%	0.72	0.24	Immediate reduction sustained over time
NSW	15	7,739	404,869	2.60	1.38	11%	7 to 16%	0.90	0.08	Linear reduction per year
SA	5	2,065	122,435	2.08	2.05	8%	1 to 15%	0.92	0.13	Linear reduction per year
TAS	3	1,007	41,850	0.90	0.68	0%	-52 to 34%	1.00	0.21	No reduction
WA	5	2,167	122,025	1.96	1.62	0%	-22 to 18%	1.00	0.17	No reduction
VIC	11	5,184	305,270	No data	No data	No data	No data	No data	No data	n/a
NT	1	335	19,667	No data	No data	No data	No data	No data	No data	n/a
Total	50	24,482	1,294,656							

QLD = Queensland, ACT = Australian Capital Territory, NSW = New South Wales, SA = South Australia, TAS = Tasmania, WA = Western Australia, VIC = Victoria, NT = Northern Territory.

* Per 10,000 bed days.

† 12 months post intervention

### Costing Information

The costs of the initiative were incurred by Hand Hygiene Australia at a national level and by the co-ordinating patient safety group in each state and territory. The estimates reflect the annual on-going maintenance costs and establishment and research costs have been excluded. Prospective surveys were used to estimate the costs of extra alcohol based hand rub and the time costs incurred by the infection control practitioners and other staff who worked in each of the 50 study hospitals to support the initiative. All costing methods are summarised in Appendix D in S1 File, a detailed description of the methods are available as are a complete set of cost results [37, 38]. All cost parameters used are summarised in Appendix E in S1 File.

Cost savings arose from reduced length of stay in ward and ICU beds because cases of *Staphylococcus aureus* bacteraemia were prevented. Estimates of the extra days saved per case of *Staphylococcus aureus* bacteraemia came from a survival model that appropriately accounts for the timing of infection during the admission [39, 40] and the results of this work are published [41]. The parameter values used are shown in Table 2. The dollar value assigned for a ward bed day was obtained from a summary of 2011–12 expenditure and activity for Australian Public Hospitals [42]. The value of an ICU bed day was obtained from a costing study of ICU admissions to a public hospital in Australia [43] and adjusted to 2011 prices based on a rate of health spending inflation [44], Table 2.

**Table 2:** Values used to estimate cost savings and years of life gained per *Staphylococcus aureus* bacteraemia avoided.

	Extra Days saved per SAB avoided (mean, se)
Ward bed, patient died	1.00 (2.60)
Ward bed, patient discharged	11.40 (2.22)
ICU bed, patient died	1.60 (0.69)
ICU bed, patient discharged	1.00 (0.56)
	Dollar value used, Minimum to Maximum
Ward bed day (min to max)	$919 to $1,252
ICU bed day (min to max)	$3503 to $4,282
	Years of life
Mean age at death (s.e.)	62.00 (0.219)
Life expectancy	69.70
Years life lost (discounted at 3%)	7.70 (6.78)

### Risks of death from *Staphylococcus aureus* bacteraemia

The extra risk of death from *Staphylococcus aureus* bacteraemia was estimated using the same survival model used to predict extra length of stay [41], and the findings are the number of deaths averted from fewer cases of *Staphylococcus aureus* bacteraemia per hospital. The analysis revealed 16.7% deaths among cases and 5.9% deaths among controls and the log of the hazard ratio was 1.27 (s.e. = 0.13). A normally distributed log relative risk was used for the cost-effectiveness modelling and the exponent used to update model results. The extra years of life gained from a death averted due to *Staphylococcus aureus* bacteraemia was the difference between the mean age at death among those who died in the sample, and life expectancy based on Australian life tables [45]. All relevant values used for the modelling are shown in Table 2.

### Model Estimation

Prior statistical distributions were fitted to each of the parameters of the model at the level of the hospital to capture uncertainty in the information used. Normal distributions were suitable for most parameters. For the cost parameters gamma distributions were appropriate to ensure costs were positive. Uniform distributions were used where no information on the shape of the distribution was available and only high and low estimates were available. The model was simulated for 5,000 random draws from all distributions, giving 5,000 estimated results for all outcome measures. The highest and lowest 2.5% for each outcome measure were used to show 95% uncertainty intervals. The probability the National Hand Hygiene Initiative is cost-effective is the proportion of 5,000 resamples with a cost per life year saved below an Australian threshold value of $42,000 per life year saved. This figure was chosen based on analysis of decisions made by the Australian government to fund new drugs from the public purse [46]. This approach to generating cost-effectiveness information is called probabilistic sensitivity analysis and has been shown to have wide applicability for health services decision-making [47–49]. A worked example and discussion of how this method applies to the economic evaluation of hand hygiene initiatives has been published [50]. The data used for the model are available, see S1 Data.

### Ethics Statement

This research was undertaken with approved ethical clearance by the University and the Hospitals’ Human Research Ethics Committees. The reference numbers for the ethical clearances are: Queensland Health. HREC/10/QPAH/180 (hospitals) and QUT HREC 1000001240 (University). For New South Wales all 15 sites were covered by this single HRECs approval. Sydney Local Health District (Concord) HREC (Ref: LNR/12/CRGH/44CH62/6/2012-038). For Victoria there were 11 sites in the study covered by 10 different HRECs: Box Hill Hospital: Eastern Health HREC (Ref: LR89/1112); Frankston Hospital: Peninsula Health Quality and Clinical Governance (Ref: HREC/12/PH/39); Geelong Hospital; Monash Medical Centre and Dandenong Hospital: Southern Health Research Directorate–Quality Assurance (Ref: 12114Q); Western Hospital: Western Health Low Risk Human Research Ethics Panel, Office for Research (Ref: QA 2012/77); The Alfred Hospital: The Alfred Ethics Committee (Ref: 217/12); St Vincent’s: Research Governance Office (Ref: LRR071/12); The Austin Hospital: Austin Health HREC, Research Ethics Unit (Ref: H2012/04672); Royal Melbourne: Melbourne Health HREC (Ref: QA 201090); The Northern Hospital: The Northern Hospital HREC Office (Ref: LR 13/12). For Western Australia there were 5 sites in the study covered by 3 different HRECs: Sir Charles Gairdner Hospital, King Edward Memorial Hospital and Princess Margaret Hospital: Sir Charles Gairdner HREC (Ref: 2011–108); Fremantle Hospital: Southern Metropolitan Area Health Service HREC (Ref: S/11/289); Royal Perth Hospital: Royal Perth Ethics Committee (Ref: RA-11/024). For Northern Territory there was only 1 site in the study from Northern Territory, Human Research Ethics Committee of Northern Territory Department of Health and Menzies School of Health Research (Ref: HREC-11-1543). For South Australia there were 5 sites in the study from South Australia, covered by 3 different HRECs: Lyell McEwin and The Queen Elizabeth Hospital were covered by SA Human Health Research Ethics Committee (Ref: 2011108); Royal Adelaide Hospital was covered by Royal Adelaide Hospital Research Ethics Committee (Ref: 110712); Flinders Medical Centre and Repatriation General Hospital were covered by Southern Adelaide Health Service Clinical Research Ethics Committee (Ref: EC00188). For Queensland there were 9 sites in the study from Queensland, covered by 2 different HRECs: RBWH, Townsville, Gold Coast, Logan, Prince Charles, Princess Alexandra, Nambour and Cairns Base were all covered by the Metro South Health Service District HREC (Ref: HREC/10/QPAH/180); Ipswich hospital was covered by reference: AU/1/3BO7013/ HREC/10/QWMS/40. For Tasmania there were 3 sites in the study covered by 1 HREC, Office of Research Services, University of Tasmania, Human Research Ethics Committee (Ref: H11999). For Australian Capital Territory there was 1 site in the study covered by ACT Health HREC (Ref: ETHLR.12.050). Written informed consent was given by participants who answered all surveys used for the study. All clinical/patient records were anonymous and de-identified.

## Results

The largest effect from the initiative was found in ACT with a 28% immediate reduction of *Staphylococcus aureus* bacteraemia infection risk that was sustained for 12 months, QLD also showed an immediate reduction of 17% of *Staphylococcus aureus* bacteraemia risk that was sustained for 12 months. There were linear reductions in NSW and SA that resulted is risks being lower at 12 months post intervention by 11% and 8% respectively. There were no discernible mean changes to infection risks in TAS and WA after the adoption of the initiative, Table 1.

**Fig 2:**
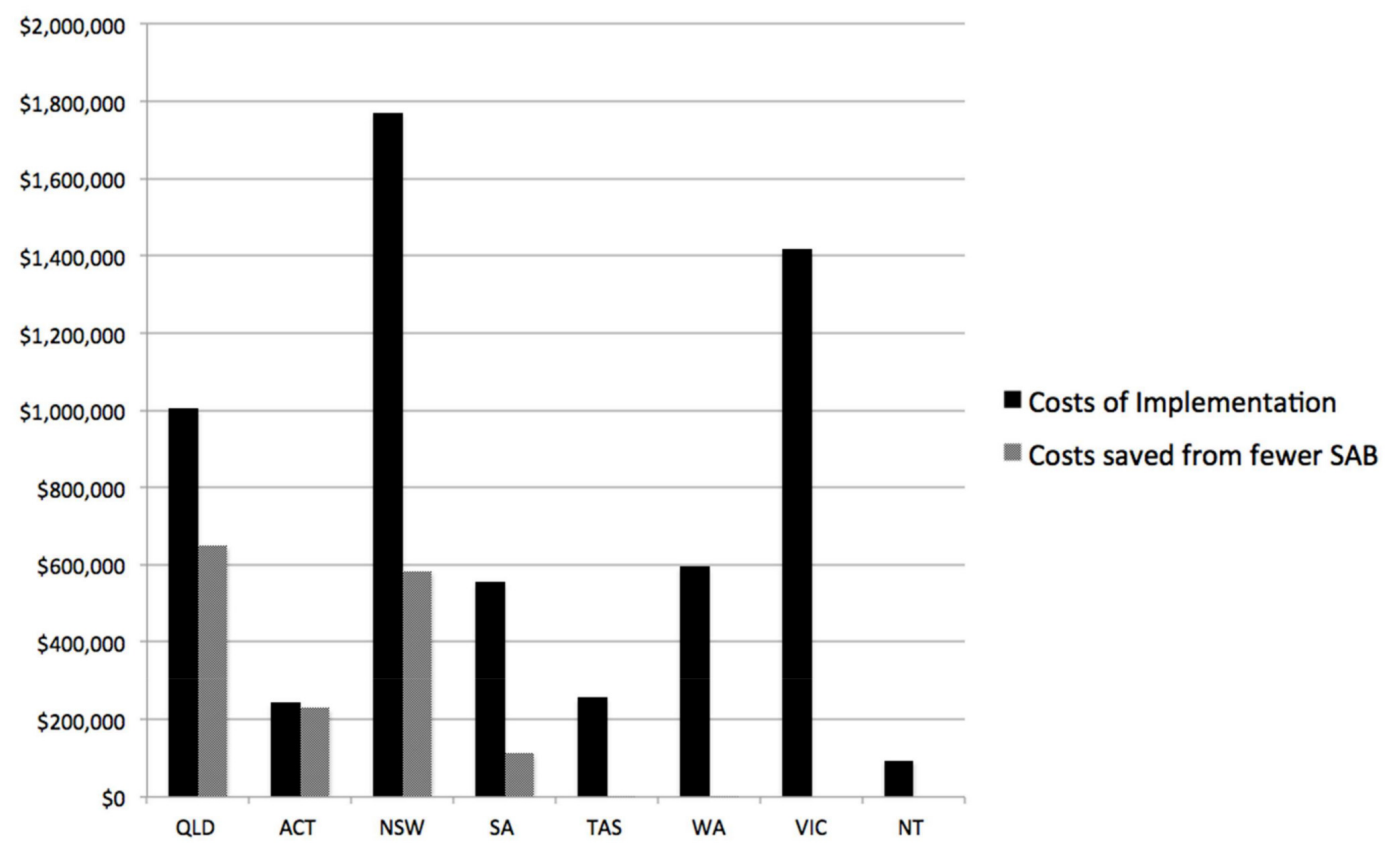
Cost increases and cost savings by state and territory for the National Hand Hygiene Initiative.

For the six states and territories that provided complete data on *Staphylococcus aureus* bacteraemia total annual costs arising from the National Hand Hygiene Initiative were $2,851,475 for a return of 96 years of life, an incremental cost-effectiveness ratio of $29,700 per life year gained. This is below the threshold for cost-effectiveness of $42,000 per life year gained used for this study [46]. As the decision to fund hand hygiene programmes lies with each state and territory we report the rest of the results at that level, Table 3 and Fig 2. The largest increases to costs were in NSW that has 7,739 beds in the study. For the ACT the estimated cost increases were small as they had only 619 beds in the study yet 9.74 cases of *Staphylococcus aureus* bacteraemia were prevented leading to relatively large cost savings. There is some chance the initiative was cost-saving in ACT as the lower uncertainty interval for the change to total costs was negative $155,769, Table 3. The number of bed days saved is reported in Appendix F in S1 File.

**Table 3:** Change to outcomes from adoption of National Hand Hygiene Initiative & Probability an Adoption Decision was Cost-Effective.

	Mean (95% Uncertainty Intervals)				
	Cases of *Staphylococcus aureus* bacteraemia prevented	Total Costs	Health Benefits in Life Years Gained	Cost per life year gained	Probability cost-effective
QLD	27.48 (23.75–31.22)	$355,344 ($166,561 - $526,681)	39.53 (28.74–52.63)	$8,988	100%
ACT	9.74 (5.08–14.43)	$14,439 (-$155,769 - $151,012)	14.01 (6.85–22.50)	$1,030	100%
NSW	24.73 (18.41–31.22)	$1,187,067 ($954,645 - $1,398,475)	35.59 (23.86–50.05)	$33,353	81%
SA	4.75 (-1.03–10.55)	$442,807 ($273,290 - $605,656)	6.84 (-1.48–15.64)	$64,729	26%
TAS	0.01 (-2.47–2.52)	$256,247 ($170,356 - $343,044)	0.02 (-3.63–3.68)	$10,371,874	1%
WA	0.01 (-6.00–6.10)	$595,471 ($441,030 - $753,395)	0.01 (-8.81–8.76)	$63,332,051	1%

QLD = Queensland, ACT = Australian Capital Territory, NSW = New South Wales, SA = South Australia, TAS = Tasmania, WA = Western Australia

* In ACT there is a 41% probability it was cost saving

There were gains to health benefits in four states, but negligible gains in TAS or WA where the initiative had no virtually no impact on *Staphylococcus aureus* bacteraemia outcomes. The initiative was cost-effective when judged against the threshold value of $42,000 per life year gained in QLD, ACT and NSW, but was not cost-effective in SA, TAS or WA, meaning that remaining with the local initiative was a better decision for the latter three states. The probabilistic sensitivity analysis showed a high probability the decision to adopt the national programme was cost effective in QLD, ACT and NSW, but a probability lower than 50% was found for the other three states and territories. This is shown explicitly by the data included in Appendix G in S1 File. The generalizability of the model results is supported by the fact that 24,482 bed-days supplied by the study hospitals accounted for 42% of all public beds in Australia, and that the number of *Staphylococcus aureus* bacteraemia included in the model for the 12 months prior to the initiative was 1,617, which represents 93% of all the cases of reported for every Australian hospital in 2010–11 [51].

### Other infection outcomes not included in cost-effectiveness model

**Fig 3:**
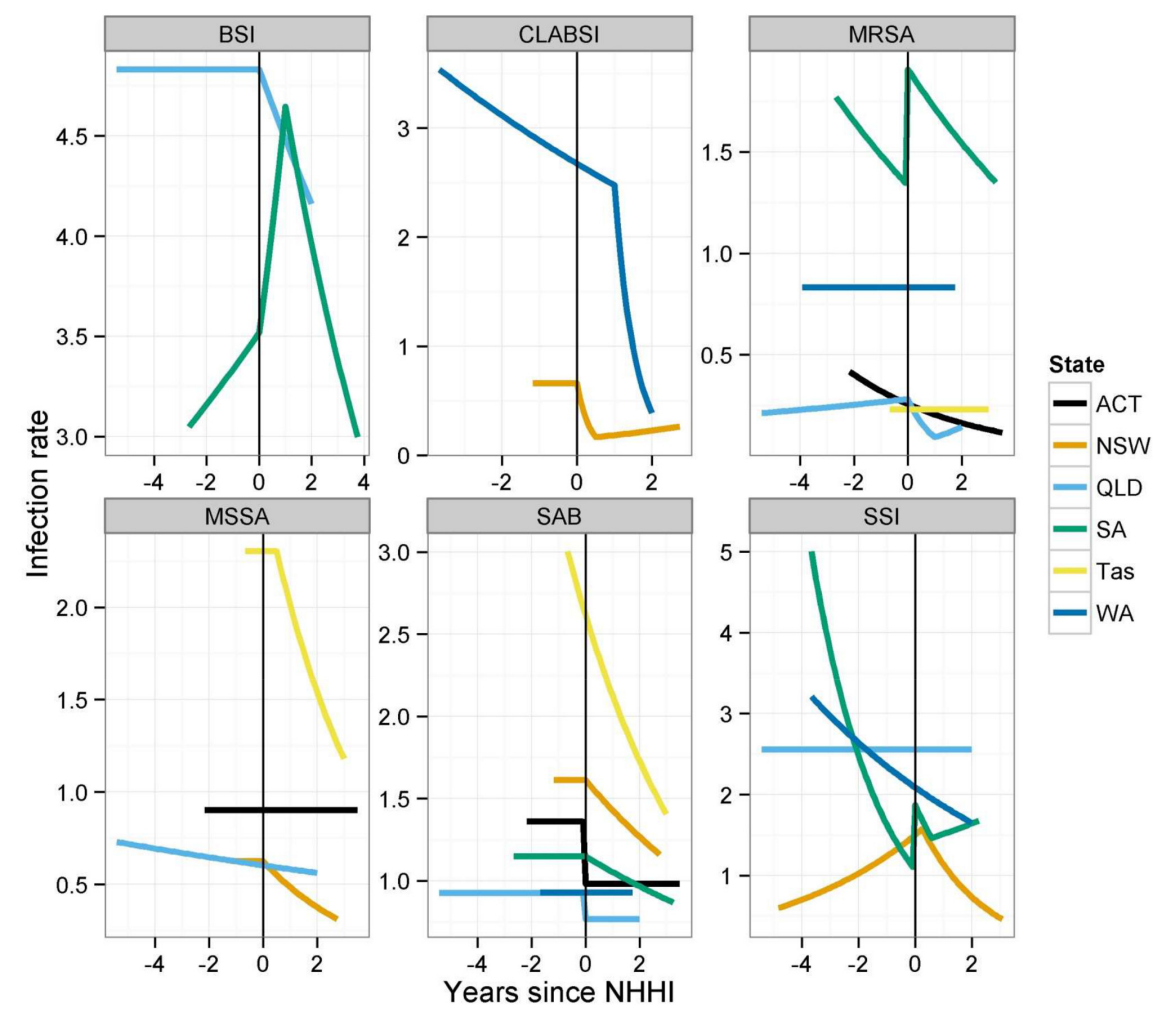
Estimated mean change in infection rates after the intervention in each state/territory. Results from the best fitting regression model in each state/territory, reproduced from Barnett *et al*. [23]

Some data were available for other infection outcomes. Rates of all bloodstream infection (BSI), central line related blood stream infection (CLABSI), methicillin resistant *Staphylococcus aureus* (MRSA), methicillin sensitive *Staphylococcus aureus* (MSSA) and surgical site infection (SSI) were obtained from all the states and territories in the study and analysed using an appropriate statistical method [23]. Not all jurisdictions collected all these outcomes and only a patchwork was available. These outcomes were not included in the cost-effectiveness model because the definitions and data collection approaches used to obtain these data are not consistently applied across the country [29] and these infection outcomes make subsets of each other. For example ‘all blood stream infections’ will include *Staphylococcus aureus* bacteraemia and CLABSI; and SSI infections will contain some MRSA and MSSA cases. The results for these other infection outcomes are reproduced from the original publication [23] in Fig 3. The National Hand Hygiene Initiative was associated with a statistically significant reduction in infection rates in 11 out of 23 state and infection combinations studied. There was no change in infection rates for 9 combinations, and there was an increase in three infection rates in South Australia.

## Discussion

This is the first cost-effectiveness evaluation of a National Hand Hygiene Initiative and shows that overall the programme was cost effective with a cost per life year gained of $29,700. It was strongly cost-effective in QLD and ACT, somewhat cost-effective in NSW but exceeded the threshold value of $42,000 per life year gained in SA, TAS and WA. The health returns and cost savings from the investment made in VIC and NT [52] are unknown as useable pre-intervention *Staphylococcus aureus* bacteraemia data were not available. This result corresponds with a recent and high quality evaluation of a decade of investment in infection prevention that showed an incremental cost-effectiveness ratio of $14,250 per life year gained ($19,479 AUD) for interventions to reduce risk of ventilator associated pneumonia and $23,277 per life year gained ($31,835 AUD) for interventions to reduce risk of central line associated blood stream infections [53].

The large variability in the cost-effectiveness results among the states and territories likely has multiple factors. Hand hygiene appears cost effective where pre intervention rates were not declining, Queensland and the ACT. Other settings such as Tasmania were on the path toward improvements regardless of the national programme and the relatively high additional cost of the national programme were not sufficiently rewarded with health benefits from fewer cases of *Staphylococcus aureus* bacteraemia. Recent research shows that hand-hygiene was only effective in time-periods where infection rates were above a critical threshold and the highest impacts where in areas of higher baseline infection rates [54]. Policy makers might conclude that the intensity of hand-hygiene campaigns be adapted to variability in local conditions. Strain dynamics might also contribute to variability in cost-effectiveness. Lawes et al. [55] showed the impact of infection prevention is strain-specific. In particular when hospital epidemiology is dominated by community associated strains, hospital based interventions have lower impact.

An important caveat is the process used to generate the effectiveness information included in the cost-effectiveness modelling [35]. As this was a national initiative there were no hospitals to act as controls and the findings are vulnerable to other changes to infection control policy. The potential for confounding is somewhat mitigated because the initiative was started at different times among the 38 study sites. This will reduce the overall correlation between the intervention and other changes. Better quality evidence would have emerged from a prospective study using a cluster randomised controlled trial or a stepped wedge design [56] as the initiative was rolled out. Yet national public health priorities often over-ride the need to do prospective evaluation with a stronger design. An opportunity to use a better design would have helped disentangle the complex effects of the likely multiple modifiable ecological factors such as antibiotic stewardship, isolation, decolonisation and improvement to hand-hygiene [57]. As with many of these projects the request to evaluate the cost-effectiveness of the initiative came after the programme had commenced making retrospective evaluation the only option. Policy makers in the future might evaluate the cost-effectiveness of a new health programme prior to national implementation.

That only *Staphylococcus aureus* bacteraemia outcomes were included is also important and means this could be a conservative estimate of the health benefits of improving hand hygiene compliance. Lawes et al [54] have shown reduced community MRSA from improving infection prevention in acute hospitals. If other infections were reduced then further cost savings and health benefits would have accrued for no extra implementation cost. The effect of this would be larger cost savings, lower total cost and higher health benefits. The patchy evidence assembled for other infection outcomes does not show significant reductions in all of the infection combinations studied. Indeed there were increases in rates of all BSI, MRSA and SSI in South Australia. This is plausible with infection control professionals in the study hospitals reporting that many of their existing tasks were displaced by the extra demands from implementing the National Hand Hygiene Initiative. Evidence would suggest that hand hygiene improvements are less effective at interrupting endogenous transmission, more likely for MSSA, than exogenous transmission, which in the hospital settings is more likely to be associated with MRSA. The English ‘clean your hands’ evaluation [5] showed no association between hand hygiene consumables use and methicillin sensitive *Staphylococcus aureus* bacteraemia. The data we had available for all MRSA, shown in Fig 3, do not however show dramatic reductions.

Only including ward and ICU bed day costs might understate the true cost savings from an avoided case of *Staphylococcus aureus* bacteraemia. It is possible that other savings occurred in the primary care sector such as interactions with community nurses, but we judge these to be a small proportion of the total costs of *Staphylococcus aureus* bacteraemia. Another related issue is that accounting costs used to value the bed days saved, $919 to $1,252 for a ward bed and $3503 to $4,282 for an ICU bed. A recent contingent valuation study elicited the economic value of a hospital bed day from a sample of 11 European hospitals [58] and found values of €72 per ward bed day and €190 per ICU bed day, much lower than the accounting costs. The impact of using lower values per bed day saved would be to dramatically reduce the cost savings and so increase the total costs of the initiative, worsening the cost-effectiveness ratios. The ‘economic’ rather than the ‘accounting’ value is the correct one to use for informing decisions about how scarce resources are used [13, 59]. Life years gained rather than quality adjusted life years gained was the measure of health benefit. Including a preference based utility decrement for the time patients spent with symptoms from *Staphylococcus aureus* bacteraemia will make a very small difference to health benefits, because the mortality benefit was for multiple years and the time in a *Staphylococcus aureus* bacteraemia state was less than two weeks. Whether the programme has matured, impacting on cost-effectiveness for time periods after 2011 to 2012 is not known, and would require new data collection.

The pressing question for policy makers is whether cheaper health returns could have been achieved by allocating scarce infection prevention budgets to programmes that were displaced by the National Hand Hygiene Initiative. This is an empirical question and requires models of the cost-effectiveness of other programmes. If the screening and isolation of in-patients, or the expansion of environmental cleaning programmes or establishing of antimicrobial stewardship programmes generated health benefits at a lower cost than improving hand hygiene, then resources should be re-allocated appropriately. The impact of this would be to increase health benefits from scarce infection prevention resources. A balanced portfolio of infection prevention activities that generates the largest health return per dollar invested is a sensible policy goal, and cost-effectiveness data are important for achieving it.

## Supporting Information

S1 File Appendix A. The source for this definition is the Australian Commission on Safety and Quality in Health Care. Appendix B. The 50 study hospitals selected for cost-effectiveness evaluation. Appendix C. Time periods for which Staphylococcus aureus bacteraemia data were available for each of the study hospitals (n = 38). Circles denote the start date of the National Hand Hygiene Initiative. Appendix D Summary of Costing Methods. Appendix E. Costing information used for modelling. Appendix F. Number of bed days saved from adoption of National Hand Hygiene Initiative. Appendix G. Uncertainty for cost-effectiveness outcomes, 1000 simulations shown for each state and territory. Results of probabilistic sensitivity analysis.
(DOCX)

S1 Data Data used for the cost-effectiveness modelling. (XLSM)
