# Supplementary material for: Cost-Effectiveness of a National Initiative to Improve Hand Hygiene Compliance Using the Outcome of Healthcare Associated Staphylococcus aureus Bacteraemia
Source: PLoS One. 2016 Feb 9;11(2):e0148190. doi: 10.1371/journal.pone.0148190 (PMC4747462; doi:10.1371/journal.pone.0148190)
Supplement: S1 File — Appendix A. The source for this definition is the Australian Commission on Safety and Quality in Health Care. Appendix B. The 50 study hospitals selected for cost-effectiveness evaluation. Appendix C. Time periods for which Staphylococcus aureus bacteraemia data were available for each of the study hospitals (n = 38). Circles denote the start date of the National Hand Hygiene Initiative. Appendix D Summary of Costing Methods. Appendix E. Costing information used for modelling. Appendix F. Number of bed days saved from adoption of National Hand Hygiene Initiative. Appendix G. Uncertainty for cost-effectiveness outcomes, 1000 simulations shown for each state and territory. Results of probabilistic sensitivity analysis. (DOCX) [file pone.0148190.s001.docx]

**Appendix A. The source for this definition is the Australian Commission on Safety and Quality in Health Care [**[**1**](#_ENREF_1)**]**

**
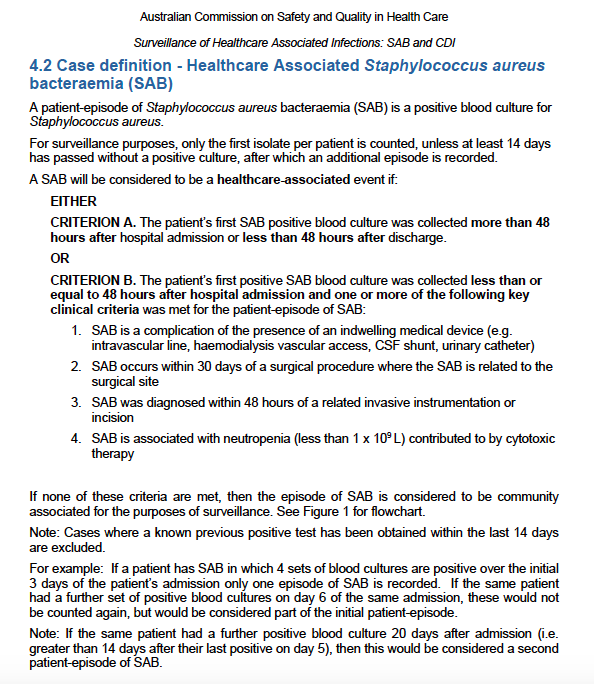
**

**Appendix B. The 50 study hospitals selected for cost-effectiveness evaluation**

| **STATE** | **HOSPITAL NAME** |
| --- | --- |
| QLD | Royal Brisbane and Women's |
| QLD | Townsville Hospital |
| QLD | Princess Alexandra Hospital |
| QLD | Cairns Base Hospital |
| QLD | Ipswich Hospital |
| QLD | Logan Hospital |
| QLD | Prince Charles Hospital |
| QLD | Nambour Hospital |
| QLD | Gold Coast Hospital |
| NSW | Gosford Hospital |
| NSW | Prince of Wales Hospital |
| NSW | Tamworth Hospital |
| NSW | Campbelltown Hospital |
| NSW | Bankstown/Lidcombe Hospital |
| NSW | St George Hospital |
| NSW | John Hunter Hospital |
| NSW | Concord Hospital |
| NSW | Royal Prince Alfred Hospital |
| NSW | Nepean Hospital |
| NSW | Westmead Hospital |
| NSW | St Vincent's Darlinghurst |
| NSW | Wollongong Hospital |
| NSW | Royal North Shore Hospital |
| NSW | Liverpool Hospital |
| WA | Fremantle Hospital |
| WA | Sir Charles Gairdner Hospital |
| WA | Royal Perth Hospital |
| WA | King Edward Memorial Hospital |
| WA | Princess Margaret Hospital |
| SA | Lyell McEwin Hospital |
| SA | Royal Adelaide Hospital |
| SA | Flinders Medical Centre |
| SA | The Queen Elizabeth Hospital |
| SA | Repatriation Hospital |
| TAS | Royal Hobart Hospital |
| TAS | Launceston General Hospital |
| TAS | North West Hospital (Burnie) |
| NT | Royal Darwin Hospital |
| ACT | The Canberra Hospital |
| VIC | Box Hill Hospital |
| VIC | St Vincent's Melbourne |
| VIC | Royal Melbourne Hospital |
| VIC | Geelong Hospital |
| VIC | The Alfred Hospital |
| VIC | Austin Hospital |
| VIC | Dandenong Hospital |
| VIC | Monash Medical Centre |
| VIC | Frankston Hospital |
| VIC | The Northern Hospital |
| VIC | Western Hospital |

**
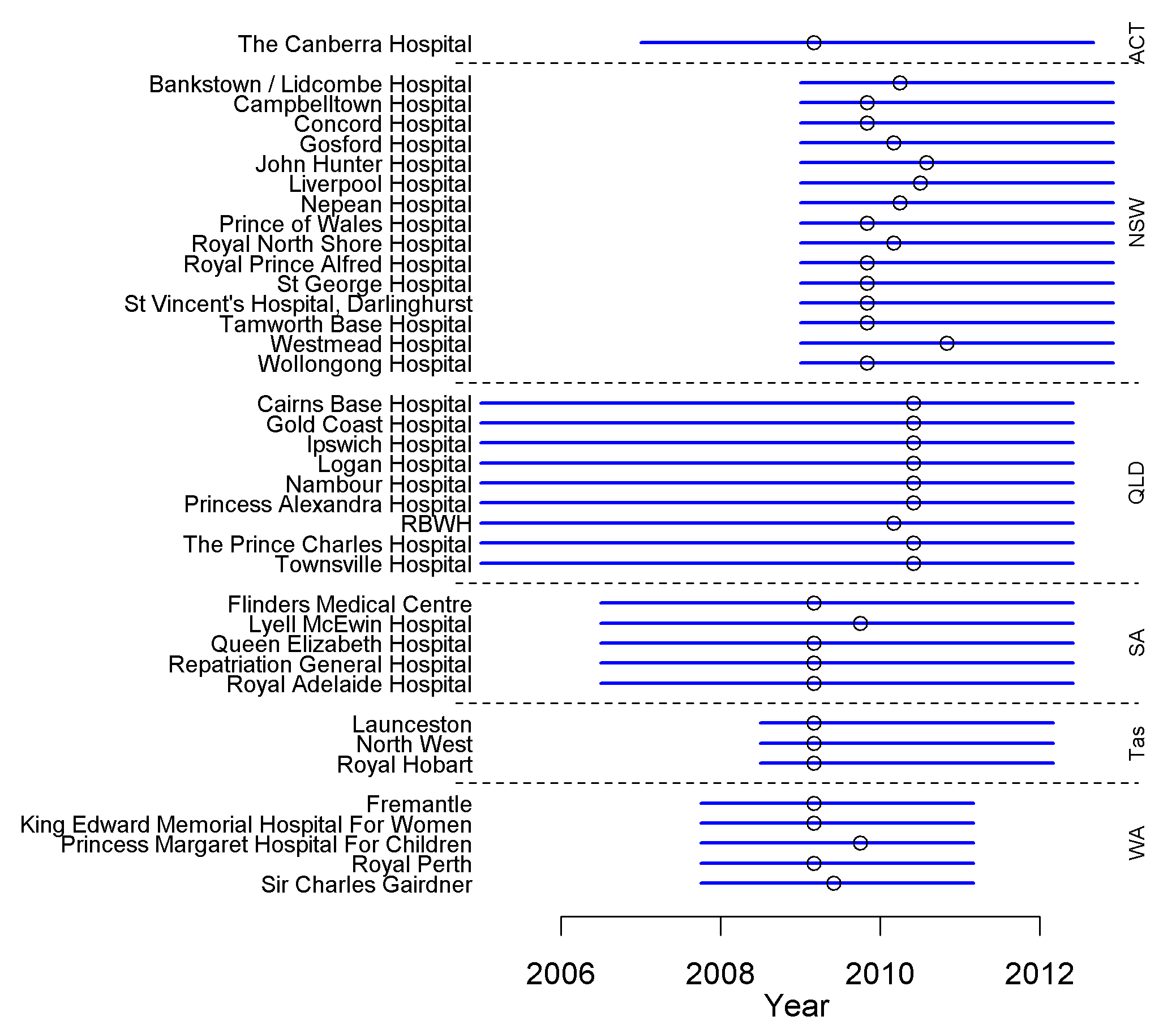
Appendix C. Time periods for which *Staphylococcus aureus* bacteraemia data were available for each of the study hospitals (n=38). Circles denote the start date of the National Hand Hygiene Initiative.**

**Appendix D Summary of Costing Methods**

The costs of the national hand hygiene initiative were incurred in four main categories. Here is a brief overview of the methods used and the full papers for methods [[2](#_ENREF_2)] and a description of the cost outcomes [[3](#_ENREF_3)] are available.

1. Time costs of hospital staff (50 study hospitals)

An online survey was administered to the senior infection control practitioner in each study hospital responsible for the implementation of the national hand hygiene initiative. The survey had been pilot tested and validated prior to roll out. Its purpose was to elicit the best estimates and measures of uncertainty for key cost parameters including the number of staff who worked on the national hand hygiene initiative, their full costs of employment, how much of their time was spent on the national hand hygiene initiative against other competing activities, and what range of activities were undertaken for the national hand hygiene initiative. Minimum and maximum values were elicited for all time estimates in order to capture the uncertainty in the estimate. A member of the research team was available for respondents to telephone if they found parts of the survey difficult. The survey could be saved, while respondents accessed diaries and time sheets, and then opened up again for their data input. Great emphasis was placed on accurately identifying only the incremental changes to costs that arose from the adoption of the national hand hygiene initiative. The final survey emerged from 3 months of methods work including semi structured interviews with infection control practitioners who had managed the programme. In particular we worked on the ways of dealing with uncertainty and overconfidence with respondents and methods for partitioning jointly used resources.

2. The administration costs of Hand Hygiene Australia who managed the initiative.

Data on the annual running costs of Hand Hygiene Australia for a three-year period were obtained. The time frame which best aligned with the other cost data used was the 2011–12 financial year. The total running costs for the relevant time period were allocated among the states based on the proportion of public beds. To allow for the negative skew in the data that describe number of beds in Australia’s public hospitals we made a judgement that 85% of the costs were used for the 50 study hospitals (the 50 largest in Australia accounting for 42% of beds). Our reasoning was that most of the Hand Hygiene Australia costs are fixed costs, for salaries and overheads, and these will not change substantially when the program is rolled out to smaller hospitals. This 85% figure was discussed and approved during a steering group meeting with Hand Hygiene Australia and the Australian Commission for Safety and Quality in Health Care. The natural interpretation of the assumption is that if the hand hygiene initiative were to be rolled out to the 50 largest study hospitals then 85% of the observed costs would be required to achieve this. The minimum estimated cost for each state was based on the percentage of public hospital beds that the study hospitals supply, and the maximum was based on all public hospital beds, including those in the long tail of the distribution.

3. The administration costs incurred by state/territory co-ordinating group

These were obtained from the relevant manager in each state and territory by an interview process. They were asked to estimate the time their staff had spent on the National Hand Hygiene Initiative, Information and Technology costs, travel costs and consumables. Respondents reported having good records of the resources used for the program over a given period of time and they were confident in their estimates. Some states had existing hand hygiene programs, so the marginal costs of switching to another program with similar attributes were low.

4. The costs of consumables including extra alcohol based hand rub

The major consumable item used was extra alcohol-based hand rub. Other consumables such as t-shirts, educational materials like posters and brochures and printing was incurred at the local hospital level and were reported in the hospital costing survey (see section 1. of this appendix). The majority of the brackets and a lot of the alcohol-based hand rubs existed or were used before the national hand hygiene initiative was implemented, therefore we only estimated the marginal costs of extra product resulting directly from the national hand hygiene initiative. Estimates were taken from 3 years of Queensland Health data, which spans all health districts. From these data we calculated a per annum bed day cost. This was done by using the number of public hospitals beds in each of the relevant health service districts and dividing the total costs for that year by the beds numbers. The fraction attributable to the changes from the national hand hygiene initiative was at a minimum (10%), average (20%), and maximum (30%). This assumption was discussed and approved at a steering group meeting and a normal distribution was assumed for these costs. Previous research investigating the pilot program of the National Hand Hygiene Initiative [19, 34, 35] has shown anywhere from an average 2 to 5 fold increase in the use of alcohol-based hand rub, measured in litres per 1000 bed days, following the introduction of the program.

**Appendix E. Costing information used for modelling**


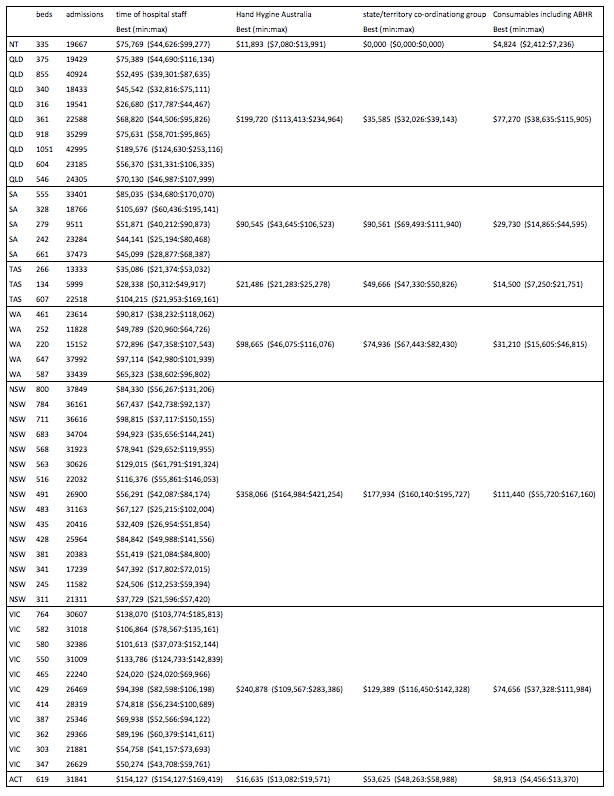


**Appendix F. Number of bed days saved from adoption of National Hand Hygiene Initiative**

|  | Mean (95% Uncertainty Intervals) | |
| --- | --- | --- |
|  | Ward bed days saved | ICU bed days saved |
| QLD | 340.84 (255.17 - 431.97) | 71.76 (50.34 - 94.61) |
| ACT | 120.38 (43.12 - 221.88) | 25.38 (7.07 - 49.88) |
| NSW | 307.11 (216.38 - 404.68) | 64.45 (44.34 - 87.71) |
| SA | 58.71 (-13.19 - 140.82) | 12.32 (-3.62 - 31.04) |
| TAS | 0.26 (-32.67 - 33.50) | 0.04 (-7.07 - 7.22) |
| WA | -0.01 (-79.82 - 78.84) | 0.05 (-16.95 - 16.90) |

**Appendix G. Uncertainty for cost-effectiveness outcomes, 1000 simulations shown for each state and territory. Results of probabilistic sensitivity analysis**


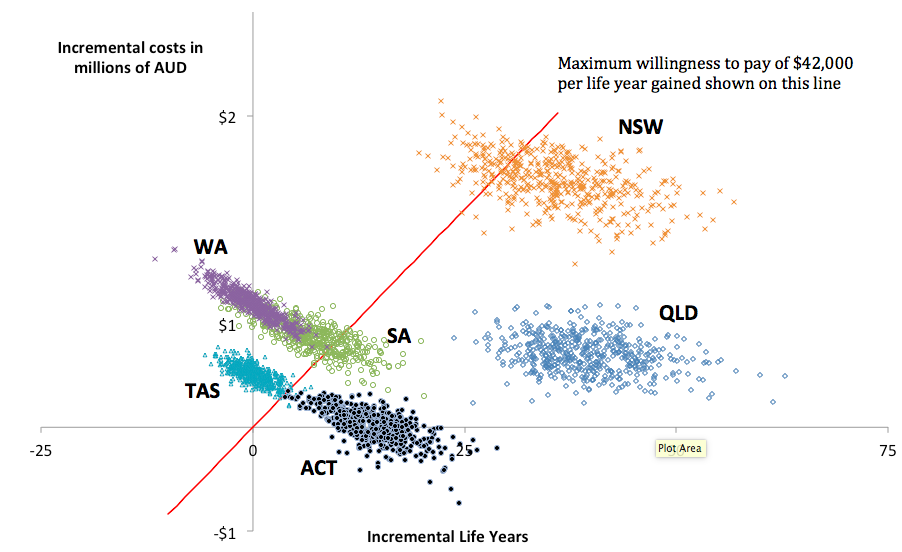


1. ACSQHC, *Surveillance of Healthcare Associated Infections: Staphylococcus aureus bacteraemia & Clostridium difficile infection. Version 4.0. Available here:* [*http://www.safetyandquality.gov.au/wp-content/uploads/2012/03/Data-Set-Specification-Surveillance-of-Healthcare-Associated-Infections-SAB-and-CDI-V-4.pdf*](http://www.safetyandquality.gov.au/wp-content/uploads/2012/03/Data-Set-Specification-Surveillance-of-Healthcare-Associated-Infections-SAB-and-CDI-V-4.pdf). 2012.

2. Page, K., N. Graves, K. Halton, and A.G. Barnett, *Humans, 'things' and space: costing hospital infection control interventions.* J Hosp Infect, 2013. **84**(3): p. 200-5.

3. Page, K., A.G. Barnett, M. Campbell, D. Brain, E. Martin, N. Fulop*, et al.*, *Costing the Australian National Hand Hygiene Initiative.* J Hosp Infect, 2014. **88**(3): p. 141-8.
